# Immunotherapy and Radiotherapy for Older Patients with Locally Advanced Non-Metastatic Non-Small-Cell Lung Cancer Who Are Not Candidates for or Decline Surgery and Chemotherapy: A Practical Proposal by the International Geriatric Radiotherapy Group

**DOI:** 10.3390/cancers16173112

**Published:** 2024-09-09

**Authors:** Nam P. Nguyen, Brandi R. Page, Huan Giap, Zineb Dahbi, Vincent Vinh-Hung, Olena Gorobets, Mohammad Mohammadianpanah, Micaela Motta, Maurizio Portaluri, Meritxell Arenas, Marta Bonet, Pedro Carlos Lara, Lyndon Kim, Fabien Dutheil, Elena Natoli, Gokoulakrichenane Loganadane, David Lehrman, Satya Bose, Sarabjot Kaur, Sergio Calleja Blanco, Alexander Chi

**Affiliations:** 1 Department of Radiation Oncology, Howard University, Washington, DC 20059, USA; satya.bose@howard.edu (S.B.); sarabjot.kaur@howard.edu (S.K.); 2 Department of Radiation Oncology, Johns Hopkins University, Baltimore, MD 21218, USA; brandi.page@gmail.com; 3 Radiation Oncology Proton Therapy, OSF HeathCare Cancer Institute, University of Illinois, Peoria, IL 61603, USA; giap@uic.edu; 4 Department of Radiation Oncology, Mohammed VI University of Health Sciences, Casablanca 82403, Morocco; dr.dahbizineb@gmail.com; 5 Department of Radiation Oncology, Centre Hospitalier Public du Cotentin, 50100 Cherbourg-en-Cotentin, France; anhxang@gmail.com; 6 Department of Oral Surgery, Cancer Tech Care Association, Perpignan 66000, France; gorobets.helen@gmail.com; 7 Colorectal Research Center, Department of Radiation Oncology, Shiraz University of Medical Sciences, Shiraz 71348-14336, Iran; mohpanah@gmail.com; 8 Department of Radiation Oncology, ASST Papa Giovanni XXIII, 24127 Bergamo, Italy; motta.micaela@libero.it (M.M.); mportaluri@asst-pg23.it (M.P.); 9 Department of Radiation Oncology, Sant Joan de Reus University Hospital, University of Rovira I Virgili, 43007 Tarragona, Spain; meritxell.arenas@gmail.com; 10 Department of Radiation Oncology, Arnau de Vilanova University Hospital, 25198 Lleida, Spain; bonet.marta@gmail.com; 11 Department of Radiation Oncology, Fernando Pessoria Canarias Las Palmas University, 35002 Las Palmas, Spain; pedrocarlos.lara@ulpgc.es; 12 Division of Neuro-Oncology, Mount Sinai Hospital, New York, NY 10029, USA; lyndon.kim@mssm.edu; 13 Department of Radiation Oncology, Clinique Sainte Clotilde, 97400 Saint Denis, France; fabiendutheil.rth@gmail.com; 14 Department of Radiation Oncology, IRCCS Azienda Ospedaliero-Universitaria di Bologna, 40138 Bologna, Italy; elenanatoli2@libero.it; 15 Radiation Oncology, Department of Medical and Surgical Sciences (DIMEC), Alma Mater Studorium, Bologna University, 40126 Bologna, Italy; 16 Department of Radiation Oncology, Institut Curie, 75005 Paris, France; gloganadane@yahoo.com; 17 Department of Radiation Oncology, International Geriatric Radiotherapy Group, Washington, DC 20001, USA; lehrman.david@gmail.com; 18 Department of Oral Maxillofacial Surgery, Howard University, Washington, DC 20059, USA; seca@umich.edu; 19 Department of Radiation Oncology, Capital University Xuanwu Hospital, Beijing 100053, China; achiaz2010@gmail.com

**Keywords:** older, frail, NSCLC, locally advanced, immunotherapy, radiotherapy

## Abstract

**Simple Summary:**

Older patients with locally advanced non-small cell lung cancer may not be candidates for standard treatment due to their poor performance status. Immunotherapy and radiotherapy are well tolerated and may become the treatment of choice for those patients. This hypothesis should be confirmed in future clinical trials.

**Abstract:**

The standard of care for locally advanced non-small-cell lung cancer (NSCLC) is either surgery combined with chemotherapy pre- or postoperatively or concurrent chemotherapy and radiotherapy. However, older and frail patients may not be candidates for surgery and chemotherapy due to the high mortality risk and are frequently referred to radiotherapy alone, which is better tolerated but carries a high risk of disease recurrence. Recently, immunotherapy with immune checkpoint inhibitors (ICIs) may induce a high response rate among cancer patients with positive programmed death ligand 1 (PD-L1) expression. Immunotherapy is also well tolerated among older patients. Laboratory and clinical studies have reported synergy between radiotherapy and ICI. The combination of ICI and radiotherapy may improve local control and survival for NSCLC patients who are not candidates for surgery and chemotherapy or decline these two modalities. The International Geriatric Radiotherapy Group proposes a protocol combining radiotherapy and immunotherapy based on the presence or absence of PD-L1 to optimize the survival of those patients.

## 1. Introduction

The standard of care for locally advanced non-metastatic non-small cell lung cancer (NSCLC) (stage III) has been neoadjuvant chemotherapy followed by surgery or surgical resection with adjuvant chemotherapy for patients with resectable tumors or concurrent chemotherapy and radiotherapy [1,2]. Recently, the addition of immunotherapy with immune checkpoint inhibitors (ICIs) following concurrent chemoradiation for unresectable tumors has attracted much attention due to improved survival but with added toxicity [3]. However, older patients, defined as 65 years of age or older, may not be surgical candidates due to co-existing morbidities and frailty [4]. Those who are frail may not tolerate chemotherapy either and are often referred for radiotherapy alone for palliation [5]. Thus, older and frail patients with locally advanced NSCLC are at risk for loco-regional recurrences and distant metastases with decreased survival. As the prevalence of lung cancer increases with age, clinicians need to devise a protocol to optimize the outcome for those patients while minimizing treatment toxicity. Even though programmed death ligand 1 (PD-L1) is not a perfect biomarker for response to immunotherapy, patients with locally advanced NSCLC with positive PD-L1 (1 or >1) are likely to benefit from ICIs [6]. Thus, any intervention that may induce a positive PD-L1 tumor response is likely to enhance its response to immunotherapy. Radiotherapy has a synergistic effect with immunotherapy and has been reported to induce a positive PD-L1 tumor formation in pre-clinical and clinical studies [7]. Immunotherapy with ICIs is also well tolerated among older patients with NSCLC [8]. Thus, combining immunotherapy and radiotherapy is a potential solution for those patients.

The International Geriatric Radiotherapy Group (http://www.igrg.org, accessed on 1 September 2024) is an organization devoted to the care of older cancer patients, minorities, and women who are frequently excluded from clinical trials [9]. Based on the currently published literature, members of the thoracic oncology cancers subgroup propose in this article a practical protocol for older patients with locally advanced NSCLC who are too frail to undergo surgery and chemotherapy or who decline those two modalities. Radiotherapy and immunotherapy may induce long-term remission and represent a potential cure for those patients.

## 2. The Role of PD-L1 in NSCLC

Programmed death ligand 1 is a transmembrane protein present in normal cells such as the gastrointestinal epithelium, dendritic cells, B cells, and macrophages to maintain immune tolerance [10]. Binding of PD-L1 to program death 1 (PD-1), a transmembrane protein present on T cells, leads to an inhibition of T cells expansion, thus preventing autoimmunity [11]. However, PD-L1 is also present on NSCLC cells in about 20–30% of cases [12]. Older age, poorly differentiated histology, EGFR wild-type, and ALK translocation are more likely to be associated with PD-L1 expression [13]. Tumors with high PD-L1 expression are frequently associated with a poor prognosis [12,14]. A high rate of nerve and blood vessel invasion, and lymph node metastasis is frequently observed. Among NSCLC patients with mediastinal lymph nodes metastases following surgery, those with high PD-L1 expression (>10%) did not respond to adjuvant chemotherapy and had a poorer survival compared to those with negative PD-L1 [15]. The 5-year survival for patients with a high PD-L1 expression was 71.1% with chemotherapy and 66.5% without. The corresponding numbers for PD-L1-negative patients were 64.1% and 48.9%. Indeed, following neoadjuvant chemotherapy for NSCLC, patients who had a higher PD-L1 level after chemotherapy were reported to be chemoresistant as they do not exhibit tumor shrinkage and had shorter survival compared to those with low PD-L1 expression [16]. Thus, the expression of PD-L1 in advanced NSCLC portends a poor outcome due to the tumor’s ability to evade the immune system and their resistance to systemic chemotherapy. Table 1 summarizes the correlation between high PD-L1 expression and poor prognosis in patients with NSCLC.

The introduction of immunotherapy with ICIs for the treatment of NSCLC in selected patients with positive PD-L1 expression has produced a significant improvement in survival for those patients associated with a substantial reduction in grade 3–4 toxicity. Among 305 patients with advanced NSCLC with a PD-L1 expression of at least 50%, randomized to pembrolizumab or cisplatin, overall survival at six months was 80.2% and 72.4% for the immunotherapy and chemotherapy, respectively [17]. The corresponding numbers for grade 3–5 toxicity were 26.6% and 53.3%. In another randomized study involving 1274 patients with advanced or metastatic NSCLC and PD-L1 > 1%, those who received pembrolizumab had a superior survival outcome compared to those treated with chemotherapy. Median survival was 20 months and 13 months for the immunotherapy and chemotherapy group, respectively [18]. The survival benefit of immunotherapy was observed for all patients with various PD-L1 levels. Corresponding grade 3 or worse toxicity was 18% and 41%, respectively. Interestingly, there is a strong correlation between PD-L1 expression and the response to immunotherapy in patients with PD-L1 50% or more. Median progression-free survival was 14.5 and 4.1 months for PD-L1 90% or more and 50–89%, respectively [19]. A meta-analysis of 10,074 patients with NSCLC corroborated the effectiveness of ICI among patients with positive PD-L1. For those who received ICI monotherapy, higher expression of PD-L1 was associated with better survival [7]. Thus, PD-L1 expression is a good biomarker to assess the response to ICI monotherapy in advanced NSCLC. Any treatment modality that increases PD-L1 expression would enhance tumor response to immunotherapy.

## 3. The Role of Radiotherapy in Increasing Tumor Cell PD-L1 Expression

A low radiation dose has been reported to increase PD-L1 expression in NSCLC cell lines in vitro [20]. An increase in PD-L1 expression was observed from 0.5% to 24.7%, 0.1% to 7.5%, and 0.1% to 11% after 2 Gy, 4 Gy, and 6 Gy in 2 Gy/fraction, respectively. Thus, low-dose conventional fractionated radiotherapy can induce PD-L1 formation in NSCLC cell lines [21]. Upregulation of PD-L1 is postulated through activation of the PI3K/AKT and STAT3 pathways [20,21]. However, the PD-L1 upregulation by low-dose radiation is blocked by PD-L1 inhibitor, leading to increase infiltration of CD8+ T cells, and inhibition of tumor growth [20]. Thus, an increase in PD-L1 expression of cancer cells following a low or conventional dose of radiation is a defense mechanism against the immune effect of radiotherapy. Other studies have investigated the effect of high radiation doses on lung cancer cell lines with doses ranging from 6 Gy times one to 6 Gy times two and 6 Gy times 3. The increase in PD-L1 expression was proportional to the radiation dose and the time elapsed since radiotherapy [22]. The surviving cells following radiotherapy exhibit high PD-L1 expression compared to parental cells and become radioresistant. An increase in Interleukin 6 (IL-6) signaling following irradiation promotes the upregulation of PD-L1 [22]. In another study, irradiation of KRAS mutant lung cancer cells implanted in mice to 17 Gy in 8.5 Gy/fraction produced an elevation of PD-L1 expression within the tumor cells associated with an increased in CD8+/Treg ratio [23]. Mice which received a combination of radiotherapy and anti-PD1 antibody had a significantly improved survival and tumor shrinkage compared to those treated with radiotherapy alone, anti-PD1 alone, or no treatment [23]. Thus, the ability of radiotherapy at a conventional or high dose to induce PD-L1 formation may serve as a strategy for clinicians to initiate radiotherapy for tumors that are PD-L1-negative to sensitize them to the effect of immunotherapy. These pre-clinical studies are conducted in the laboratory in a rigorous fashion and serve as templates for clinical studies.

Clinical studies also support the role of radiotherapy to induce PD-L1 formation in NSCLC. Yoneda et al. [24] reported the PD-L1 expression of 41 patients with stage II-III NSCLC who underwent neoadjuvant chemoradiation (*n* = 23) or chemotherapy (*n* = 18) before surgical resection. Compared to the biopsy specimens, the resected tumors of patients who underwent chemoradiation exhibits a significant increase in PD-L1 expression (91.3%) which was not observed among the ones who received chemotherapy only. Thus, the increase in PD-L1 expression was attributed to the effect of radiotherapy on the tumor cells. In addition, there was also an increase in CD-8+ T cells within the tumor stroma which paralleled the situation observed in vivo. Other studies also corroborated increased PD-L1 expression following radiotherapy for NSCLC. Adams et al. [25] reported the results of liquid biopsy of 35 patients with NSCLC. Circulating tumor cells (CTCs) were collected before and following radiotherapy. Following radiotherapy, the CTCs of 11 patients who had low or negative PD-L1 CTCs became highly expressed (31.4%). This increase in PD-L1 expression after radiotherapy for NSCLC has important clinical implications for patient survival and for response to immunotherapy. Those who had an increase in PD-L1 expression after chemoradiation for NSCLC had a poorer survival compared to the ones who were negative [26]. On the other hand, for patients who receive immunotherapy following radiotherapy for NSCLC, an increase in PD-L1 expression is a hallmark for better survival. Even though these clinical studies only included a small number of patients, they corroborated the finding that radiotherapy did increase PD-L1 expression in NSCLC.

Moran et al. [27] compared the outcome of 82 patients with recurrent or metastatic NSCLC who received immunotherapy (*n* = 41) or other therapy (*n* = 41). All patients had previous radiotherapy. Liquid biopsy was performed before and after treatment. Patients who had an upregulation in CTC PD-L1 expression after treatment experienced significant improvement in survival and progression-free survival when they received immunotherapy compared to the ones in the control group. Thus, the study demonstrates the proof of concept that the initiation of radiotherapy in NSCLC may lead to a better response to ICIs through the induction of PD-L1 formation in tumor cells. Further prospective studies need to validate this concept. Table 2 summarizes the potential of radiotherapy to enhance PD-L1 expression in NSCLC.

## 4. Efficacy and Tolerance of Older Cancer Patients with Locally Advanced NSCLC to Immunotherapy with ICI

Compared to systemic chemotherapy, immunotherapy with ICIs has a better safety profile especially for older patients due to reduced bone marrow reserve and kidney function. In a randomized study comparing first-line atezolizumab monotherapy (*n* = 302) with single-agent chemotherapy (*n* = 151) for 453 patients with stage III and IV NSCLC ineligible for platinum-based doublet chemotherapy due to their age (70 or older) and pre-existing comorbidities or poor ECOG status (2 and above), survival was significantly superior for the immunotherapy group with less grade 3–4 toxicity. The 2-year survival was 24% and 12% for the atezolizumab and chemotherapy group, respectively [28]. Corresponding grade 3–4 toxicity was 16% and 33%, respectively. Thus, monotherapy with ICIs is well tolerated and superior to chemotherapy for older patients with advanced NSCLC.

The safety profile for older patients with NSCLC was also corroborated in other studies. In a cohort of 188 patients with NSCLC who received nivolumab following relapse from previous chemotherapy, grade 3–4 toxicity was 8% and 4.8%, for patients 70 years of age or older (*n* = 38) and younger patients (*n* = 150), respectively [29]. Median survival was 14.8 months and 12.8 months for the older and younger group, respectively.

In another study, there was also no difference in safety or efficacy for older patients (70 years of age or older) (*n* = 169) who received pembrolizumab as first-line therapy for advanced or metastatic NSCLC compared to younger ones (*n* = 158). Median survival was 11.3 months and 11.2 months for older and younger patients, respectively. Acute toxicity was 26% for both groups [30]. Many studies also reported that old age has no impact on survival or toxicity for patients treated with ICI for NSCLC [31,32,33,34,35]. Thus, immunotherapy with ICI has a good safety profile among older patients with NSCLC and may have a survival benefit compared to chemotherapy. However, older patients frequently receive polypharmacy which may impact on treatment toxicity or efficacy and need to be monitored by a team familiar with geriatric management such as geriatricians to identify potential issues which may arise during their treatment [36]. In addition, the impact of frailty on treatment has not been fully investigated in current clinical trials for NSCLC patients receiving immunotherapy and needs to be incorporated in future clinical trials [37]. As ICI agents have a long half-life, increasing the interval between their administration may improve tolerance to treatment without decreasing treatment efficacy [38]. Such a strategy may be adopted if excessive toxicity occurs at the discretion of the investigator. Table 3 summarizes the efficacy and toxicity of immunotherapy for older patients with NSCLC.

## 5. Preliminary Study of Immunotherapy Combined with Radiotherapy for Locally Advanced NSCLC

A preliminary study of immunotherapy with concurrent radiotherapy is encouraging. Tachihara et al. [39] reported the results of 35 patients with stage III NSCLC (*n* = 26) or recurrent disease after surgery (*n* = 9) with a median age of 72 (range: 44–83) treated with durvalumab 10 mg/kg every two weeks for up to 12 months until disease progression or development of unacceptable toxicity combined with radiotherapy. Radiotherapy commenced on day 1 of immunotherapy to a total dose of 60 Gy in 2 Gy/fraction. All patients were PD-L1-positive (1% or more). The 1-year progression-free survival (PFS) and median PFS was 72.1% and 25.6 months, respectively. The overall response rate (RR) was 90.9% with a complete response (CR) rate of 33.3%. These results compared favorably to those reported by the Pacific trial where patients received durvolumab after chemoradiation [40]. The 1-year PFS and RR was 55.9% and 28.4%, respectively. However, in contrast to the Pacific trial grade 3–4, and grade 5 toxicity was higher at 52.9% and 5.9%. The two patient deaths in the study were due to lung infection from steroid administration (*n* = 1) and bronchoesophageal fistula following disease recurrence (*n* = 1). Grade 3–4 pneumonitis was 11.8%. Inclusion of patients with postoperative recurrence may have been a factor for the higher toxicity due to the large radiotherapy field required to cover the gross mediastinal lymph nodes. Nevertheless, the study highlights the fact that immunotherapy and radiotherapy may lead to a higher response rate and PFS in patients with locally advanced NSCLC who are PD-L1-positive.

Treatment toxicity may be mitigated by extending the interval of durvalumab administration to every four weeks instead of every two weeks. Furthermore, treatment toxicity may be further reduced to treat the area of gross disease only with a high dose as illustrated by the technique of stereotactic body radiotherapy (SBRT) for early-stage or metastatic NSCLC. Another potential alternative to reduce serious toxicity is the induction of immunotherapy to reduce the size of the gross tumor disease volume followed by radiotherapy to reduce the volume of normal lung receiving a high dose of radiation. For instance, among patients with PD-L1-positive NSCLC (1% or more), atezolizumab 1200 mg every three weeks until disease progression or unacceptable toxicity has produced a response rate of 31% observed on CT scan at regular intervals.

Patients with high PD-L1 expression (50% or more) exhibited a significant response compared to those with lower PD-L1 expression, with response rates of 40.2% and 33.7%, respectively [41]. Other studies reported response rates of 26% and 44.8% to nivolumab and pembrolizumab, respectively, for PD-L1-positive NSCLC [17,42]. The higher response rate to pembrolizumab was attributed to a selection of patients with high PD-L1 expression in the study [17]. Thus, patients with positive PD-L1 expression may benefit from induction immunotherapy prior to radiotherapy to minimize treatment toxicity.

## 6. Efficacy and Toxicity of Radiotherapy at High Doses and Immunotherapy for Early-Stage and Metastatic NSCLC

Efficacy and toxicity of high-dose radiotherapy for early-stage and metastatic NSCLC have shown promising results. The advancement of high-precision radiotherapy with image-guided techniques has improved the survival of patients with early-stage NSCLC who are not candidates for surgery due pre-existing co-morbidities. A high dose of radiation delivered through SBRT has produced high survival rates comparable to lobectomy, with minimal toxicity among older cancer patients with early-stage NSCLC [43]. However, despite achieving excellent local control rates, patients may develop distant metastases following SBRT. Immunotherapy may offer further improvement in survival by targeting micro-metastases [44]. In a study by Chang et al. [45], involving 156 patients with stage I and II, those treated with SBRT alone or combined with nivolumab 480 mg every four weeks for four cycles concurrently with radiation exhibited a 4-year PFS of 77% and 53% for the combined modality group and the SBRT-alone group, respectively. Approximately 15% developed grade 3 adverse events related to the administration of nivolumab, with no instance of grade 3 pneumonitis. These findings suggest that high radiation doses are safe when combined with immunotherapy for the treatment of NSCLC.

The benefits of radiotherapy combined with immunotherapy were also reported in patients with locally advanced or metastatic NSCLC. Li et al. [46] reported the survival and PFS of 259 patients with stage III and IV NSCLC treated with sintilimab alone (*n* = 140) or combined with radiotherapy (*n* = 119). The median survival and PFS were 30 months and 9 months, and 16 months and 5 months for the combined treatment group and the immunotherapy alone group, respectively. There was no significant difference in toxicity between the two groups with grade 3 toxicity reported in 1.7% and 2.9% of patients for the combined modality group and immunotherapy group, respectively. In another study, the addition of radiotherapy to immunotherapy did not increase toxicity for patients with metastatic NSCLC, although patients with negative PD-L1 seemed to benefit the most from radiotherapy. Median PFS for those patients was 20.1 months and 4.6 months with and without radiotherapy, respectively [47]. Thus, a high dose of radiotherapy prior to immunotherapy may induce PD-L1 formation in cancer cells, making them more vulnerable to ICIs. The survival advantage of treatment with SBRT prior to immunotherapy for patients with negative PD-L1 and advanced NSCLC was also corroborated in another study and merits further investigation [48].

A meta-analysis of 2027 NSCLC patients enrolled in 20 studies reported that the combination of immunotherapy and radiotherapy produced superior survival and PFS compared either modality alone, with no significant increase in grade 3 toxicity [49]. Even though the meta-analysis included a small number of patients and had only two randomized trials, it corroborated the safety and efficacy of the combined treatment. Thus, a protocol combining immunotherapy and radiotherapy, based on the presence or absence of PD-L1, may benefit older patients with locally advanced NSCLC who are not candidates for chemotherapy or decline this modality as part of their treatment. Table 4 summarizes the benefits of adding ICI to radiotherapy for the treatment of NSCLC.

## 7. Safety and Efficacy of Hypofractionated Radiotherapy for Older Patients with Locally Advanced NSCLC

Even though radiotherapy alone is less effective for local control and survival for patients with locally advanced NSCLC compared to chemoradiation, it does offer good palliation to improve their quality of life [6]. Among older patients, hypofractionated radiotherapy offers a significant advantage due to a shorter course of treatment in patients with reduced mobility and difficulty with transportation [9]. Patients selected for hypofractionated radiotherapy alone are often frail and have multiple co-morbidities which preclude them from having surgery and chemotherapy. Radiotherapy dose ranges from 38 to 60 Gy in 2.5 to 5 Gy/fraction [50,51,52,53,54,55,56,57].

Overall, hypofractionated radiotherapy is safe and well tolerated among older patients with locally advanced NSCLC when the fraction size remains below 4 Gy. Grade 3–4 toxicity ranges from 3.9 to 5.2% in those studies [50,53,54]. However, when the fraction size increases to 5 Gy to improve loco-regional control, unacceptable toxicity develops. Tekatli et al. [57] reported the results of 47 patients with centrally located lesions treated to a total dose of 60 Gy in 5 Gy/fraction with 15% developed grade 5 toxicity, mostly from fatal hemorrhage. The close proximity of the tumor to the large vessels likely plays a role in the development of fatal complications. Another study reported a 4% grade 5 toxicity with a fractionation of 60 Gy in 4 Gy/fraction [51]. Thus, it seems prudent to use a fractionation of 60 Gy in 3 Gy/fraction, which provides a BED of 78 Gy when developing a protocol for older patients, with locally advanced NSCLC [55]. The high rates of distant metastases (up to 60.6%) observed highlight the need to incorporate systemic therapy with hypofractionated radiotherapy instead of increasing radiation dose to improve survival.

Interestingly, in the study reported by Kravutski et al. [53], patients who recurred after radiotherapy were salvaged with immunotherapy with no added toxicity, suggesting that sequential hypofractionated radiotherapy and ICI is feasible. However, the efficacy of the treatment was not reported. Table 5 summarizes relevant studies of hypofractionated radiotherapy alone for older patients with locally advanced NSCLC.

## 8. Inclusion of Frailty in the Treatment of Older Patients with NSCLC

The inclusion of frailty is imperative in any prospective trial involving older cancer patients. A meta-analysis of patients with lung cancer revealed elevated mortality and treatment toxicity rates among frail individuals, irrespective of treatment [58]. Indeed, among 1020 patients aged 60 or older, frailty is a reliable index to predict grade 3–5 toxicity after the first cycle of chemotherapy [5].

Frailty, characterized by increased vulnerability of older adults to stressors due to age-related declines in physiologic reserves across multiple organs system [59]. While frailty can manifest at any age, it is more prevalent in older patients [60]. Assessment of frailty often involves questionnaires, as consensus on biomarkers is lacking [61]. Among the various assessment tools, the G-8 questionnaire stands out for its simplicity and efficiency in a clinical setting [62]. Patients scoring 15 or above are categorized as fit, while those scoring of 14 or less undergo a complete geriatric assessment with the comprehensive geriatric assessment (CGA) survey [63].

## 9. The Role of Liquid Biopsy in the Management of Older Patients with Locally Advanced NSCLC

Liquid biopsy offers a less invasive means of detecting tumor-derived material present in various bodily fluids, including blood, urine, saliva, and cerebrospinal fluid [64]. In the context of NSCLC, several blood-based biomarkers have been proposed: cell-free DNA (cfDNA), circulating tumor cells (CTCs), exosomes, epigenetic signatures, microRNA (miRNA) and the T-cell repertoire. The most frequently used biomarkers are cfDNA, and CTCs [65]. A notable advantage of liquid biopsy is its capability to monitor tumor response to treatment in real time. The decrease or disappearance of these biomarkers during treatment often signifies a favorable response, preceding radiographic changes [66,67,68]. Conversely, an increase in biomarker levels or their detection after being undetectable predicts disease relapse and poorer survival [69,70]. Thus, clinicians can make treatment decisions based on the dynamic of these biomarkers, potentially switching to alternative therapies as needed. Despite its limitations, liquid biopsy is comparably accurate to tumor biopsy and can complement the latter to mitigate sampling errors stemming from tumor heterogeneity [71]. Moreover, in cancer patients who undergo immunotherapy, the phenomenon of pseudoprogression—manifested by transient tumor enlargement observed on CT scans—may mimic disease progression leading to unnecessary biopsies or erroneous management decision. Although the mechanism of pseudoprogression is unclear, it is believed to involve the infiltration of CD-8 T cells induced by immunotherapy, initially leading to tumor size increase followed by subsequent shrinkage due to tumor cells death [72]. While pseudoprogression is rare, its recognition is crucial as it has been associated with favorable survival outcomes and can be potentially be diagnosed using liquid biopsy [73,74]. Consequently, the integration of liquid biopsy into immune oncology trials is essential for assessing tumor response throughout and following treatment, as the patient may achieve long-term cancer remission even after discontinuing immunotherapy early [75,76].

## 10. Future Research on Immunotherapy for Older Patients with Locally Advanced NSCLC

Even though there was no significant difference in toxicity between younger and older cancer patients receiving ICIs, the management of older patients requires a team approach due to the coexistence of multiple comorbidities, polypharmacy, a higher prevalence of depression, and social isolation. Coordination between medical and radiation oncologists, geriatricians, psychologists, patient navigators, social workers, and nursing staff is essential to optimize patient care, particularly in the coronavirus disease 2019 (COVID-19) era. Telemedicine may play a crucial role in the future as it may be a cost-effective technology to minimize physicians’ office visits in patients with limited mobility and reducing exposure to the virus. Preliminary studies suggest that telemedicine may provide a one-stop shop for older cancer patients who need systemic therapy [77,78].

Monitoring the side effects of immunotherapy and providing proper intervention when these side effects develop remain key to the success of any cancer treatment program. The use of telemedicine may enable rapid intervention by a multidisciplinary team (oncologists and subspecialists) when suspected side effects from ICIs are identified [79]. Selected side effects from ICIs, such as pituitary insufficiency, may be difficult to diagnose and require real-time input from an endocrinologist.

On the other hand, grade 3–4 of immunotherapy may be further reduced by ICI dose reduction and/or an increased interval between doses. The pharmacokinetics of ICIs differ from other chemotherapeutic agents due to a rapid saturation of the receptors, leading to an early plateau of the dose–response curve. Thus, a lower dose may achieve the same therapeutic goal as the recommended dose by the US Food and Drug administration (FDA) [80]. Preliminary evidence suggests that an alternative dosing regimen of ICIs may be effective not only for minimizing side effects but also for reducing treatment cost. For example, a randomized trial of low-dose nivolumab 20 mg every three weeks combined with chemotherapy showed improved survival for patients with recurrent or locally advanced head and neck cancer compared to those treated with chemotherapy alone [81]. In another real-world study, nivolumab at a low dose of 20 mg or 100 mg every two weeks, based on the patient financial affordability, was effective at improving survival for patients with advanced hepatocellular carcinoma without any grade 3–4 toxicity [82]. Patients who received the 20 mg dose had superior progression-free survival compared to those who received 100 mg. Those two studies provided proof of principle that low-dose immunotherapy may be effective with minimal toxicity. Other studies also corroborated the efficacy of low-dose ICIs. For example, pembrolizumab 100 mg every three weeks for advanced NSCLC produced a similar survival outcomes compared to pembrolizumab 200 mg every three weeks [83]. Thus, future prospective studies for older cancer patients treated with immunotherapy should take into consideration that adjusting the dose and/or interval of ICI administration may be necessary if excessive toxicity develops during the treatment.

## 11. Special Issues of Older Patients Monitoring during Immunotherapy

Even though the side effects of immunotherapy are less severe compared to chemotherapy, grade 3–5 toxicity may occur and require special vigilance from the clinicians. Many guidelines have been published based on expert opinions but, due to the paucity of data on older cancer patients, the recommendations are rapidly evolving.

First, it is important to highlight that the side effects of immunotherapy may be correlated to its efficacy. Preliminary data suggests that older cancer patients who developed immune-related adverse events (irAEs) may experience better survival and progression-free survival which may be treated safely with corticosteroids [84,85]. As an illustration, among 34 patients 75 years of age or older who had treatment discontinued due to irAEs, survival was significantly better compared to the ones who did not have irAEs (*n* = 103) [86]. Thus, at least for older cancer patients, clinicians should not fear the side effects of immunotherapy as they may be associated with a better response during treatment.

Second, even though irAEs can occur in any organs of the body, the prevalence of skin toxicity is more frequent in older patients compared to younger ones [87,88,89]. Older patients with a history of depression may be prone to develop irAEs and merit special attention during treatment [89]. Those who are frail may develop more frequent side effects and treatment interruption compared to fit patients [90,91]. Thus, a dose reduction may be considered for frail patients to reduce the frequency of the side effects.

Finally, regardless of age, patient sex and ethnicity may influence survival and/or side effects. Women may experience more irAEs side effects compared to men due to a difference in hormonal milieu which leads to drugs having a longer half-life [92]. Selected studies have reported a higher risk of pneumonitis among Asians receiving immunotherapy [93,94]. Future prospective studies should take into consideration ethnicity and sex difference in the study design.

## 12. Protocol of the IGRG for Older Patients with Locally Advanced NSCLC Unfit for Surgery and Chemotherapy or Who Decline Those Two Treatment Modalities

Prior to treatment, older patients defined as 65 years old or above will be screened for frailty with the G-8 and CGA questionnaires. Tumor biopsy specimens should undergo next generation sequencing (NGS), including PD-L1. Liquid biopsy with cfDNA will be performed concurrently with tumor biopsy to establish a baseline level and will be repeated monthly during and after treatment to assess tumor response to immunotherapy [95]. Patients with targetable mutations such as EGFR or ALK translocation will be excluded from the study as they typically exhibit excellent response and improved survival with targeted agents, which may not respond to immunotherapy [96,97]. Patients with negative PD-L1 (less than 1) will undergo intensity-modulated-based image-guided radiotherapy first, with a total dose of 60 Gy in 3 Gy/fraction to the gross tumor volume (GTV) and enlarged lymph nodes. Immunotherapy will commence every three weeks after radiotherapy for eight cycles. Patients with positive PD-L1 (1 or more) will receive immunotherapy first for four cycles every three weeks, followed by radiotherapy with the same dose and fractionation. Immunotherapy will resume after radiotherapy for four more cycles unless excessive toxicity is observed during the induction phase. The dose or interval of ICI administration may be adjusted by the investigators to minimize treatment toxicity. The impact of age, frailty, sex, polypharmacy, and ethnicity on outcomes will be analyzed. The study should include a large number of patients to allow stratification by age, sex, ethnicity, and frailty. The data generated may serve as a template for future prospective studies. Table 6 summarizes the IGRG protocol for immunotherapy and hypofractionated radiotherapy. With a network of 1282 institutions across 127 countries, the IGRG is committed to conducting these trials when funding becomes available [98,99].

As an illustration of the proposed protocol, an 80-year-old male with a PD-L1-negative locally advanced NSCLC not eligible for chemotherapy or targeted therapy would undergo radiotherapy to a total dose of 60 Gy in 3Gy/fraction to the tumor bed. If the patient is fit as determined by the G8 screening test he would receive a full dose of immunotherapy every three weeks for eight cycles unless significant irAEs have developed. However, if the patient is frail, the patient would receive a reduced dose of immunotherapy to minimize treatment toxicity. If the same patient has a PD-L1-positive tumor, he would receive four cycles of immunotherapy first at full or reduced dose depending on his frailty status. Radiotherapy will be initiated following immunotherapy with the same radiation dose followed by four cycles of immunotherapy if there was no serious toxicity during the immunotherapy induction phase.

## 13. Conclusions

Management of locally advanced NSCLC for older patients is a challenge due to the high rate of recurrences if chemotherapy is omitted due to frailty or patient refusal. The use of PD-L1 as a biomarker may help clinicians develop a strategy to optimize the synergy between immunotherapy and radiotherapy while minimizing treatment toxicity. Prospective studies are needed to confirm this hypothesis.

## Figures and Tables

**Table 1:** Correlation between increase PD-L1 expression and poor prognosis in NSCLC.

Study	Prognosis	Comments
Pawelczyk et al. [12]	Increase risk of mediastinal lymph node involvement, poorly differentiated histology, and poor survival	Retrospective study
		Large number of patients (*n* = 866)
Zhao et al. [14]	Increase risk of nerve or blood vessel invasion and mediastinal lymph node invasion	Retrospective study
		Small number of patients (*n* = 97)
Eichhorm et al. [15]	No benefit of chemotherapy among patients who were PD-L1-positive	Retrospective study
		Small number of patients (*n* = 277)
Zhang et al. [16]	Poor response to neoadjuvant chemotherapy and poor survival among patients with high PD-L1 expression	Retrospective study
		Small number of patients (*n* = 92)

PD-L1: programmed death ligand 1; NSCLC: non-small-cell lung cancer.

**Table 2:** Pre-clinical and clinical studies highlighting the effect of radiotherapy on PD-L1 expression in NSCLC.

Study	Findings	Comments
Wan et al. [20]	Increase in PD-L1 expression in NSCLC cell lines A549 and LLC following radiation	rigorous in vitro study, no bias
Gong et al. [21]	Increase in PD-L1 expression in NSCLC cell lines A549, PC9, and H20 after radiation	rigorous in vitro study, no bias
Shen et al. [22]	Increase in PD-L1 expression in NSCLC cell lines A547 and H-157 proportional to radiation dose	rigorous in vitro study, no bias
Herter-Sprie et al. [23]	Increase in PD-L1 expression of KRAS NSCLC implanted in mice after radiation	rigorous in vivo study, no bias
Yoneda et al. [24]	Increase in PD-L1 expression following neoadjuvant chemoradiation in patients with locally advanced NSCLC	small number of patients (*n* = 23), retrospective study
Adams et al. [25]	Increase in PD-L1 expression in CTCs after RT alone or chemoradiation in patients with NSCLC	small number of patients (*n* = 41), prospective study
Moran et al. [27]	Increase in PD-L1 expression in CTCs and circulating stromal cells after radiation in patients with recurrent or metastatic NSCLC	small number of patients, prospective study

NSCLC: non-small-cell lung cancer; PD-L1: programmed death ligand 1; CTC: circulating tumor cells.

**Table 3:** Efficacy and tolerance of immunotherapy in older patients with NSCLC.

Study	ICI	Age	Survival	Toxicity	Comments
Lee et al. [28]	atezolizumab	70 or older	24% (ICI)	16% gr. 3–4	well-designed randomized study
			12% (C)	33% gr. 3–4	
Ron et al. [29]	nivolumab	70 or older	NS	8% gr. 3–4	small number of patients (*n* = 38)
					retrospective study
					subgroup analysis
Grosjean et al. [30]	pembrolizumab	70 or older	12.7% (<70)	26% gr. 3–4	retrospective study
			12.4% (70+)	26% gr. 3–4	subgroup analysis
Imai et al. [31]	pembrolizumab	75 or older	74% disease control	15% gr. 3–4	retrospective study
				4% gr. 5	small number of patients (*n* = 47)
					short follow up (median: 10 months)
Gomes et al. [32]	various	70 or older	NS	12.9% gr. 3–5 (<70)	prospective longitudinal study
				18.6% gr. 3–5) (70+)	small number of patients (*n* = 140)
Wu et al. [33]	various	65 or older	improved survival for younger (<65) and older (65+) patients	NS	meta-analysis of 11,157
					Tumor heterogeneity as all tumors
					types were included
Marur et al. [34]	various	65 or older	14.5 months (<65)	47% gr. 3–4 (<65)	retrospective study
			14.2 months (65+)	49% gr. 3–4 (65+)	
Corbaux et al. [35]	various	70 or older	no survival	11% gr. 3–4 (<70)	retrospective study
			difference	12% gr. 3–4 (70+)	tumor heterogeneity
			based on age		as all tumor types were included

ICI: immune checkpoint inhibitor; C: chemotherapy; NS: not specified, gr: grade.

**Table 4:** Clinical studies outlining the benefits of immunotherapy and radiotherapy for NSCLC.

Study	ICI	Survival Benefit	Comments
Chang et al. [45]	nivolumab	77% (SBRT + ICI)	Randomized study
		53% (SBRT)	Small number of patients (*n* = 156)
Li et al. [46]	sintilimab	30 months (RT + ICI)	Retrospective study
		16 months (RT)	
Geng et al. [49]	various	Improved survival and progression-free	Meta-analysis
		survival of immunotherapy and radiotherapy	Only two randomized trials among
		compared to either therapy alone	the 20 studies selected
			Small number of patients (*n* = 2027)

ICI: immune checkpoint inhibitor; SBRT: stereotactic body radiotherapy; RT: radiotherapy.

**Table 5:** Hypofractionated radiotherapy for older patients with locally advanced non-small-cell lung cancer.

Study	Patient No	Age (Median)	Radiotherapy Dose	LC	RC	DM	Survival	Complications	Follow-Up (Median)
Lee et al. [50]	53	80	45 Gy	89.6%	80%	4.7%	13 m	No	NS
			3 Gy/fr				(median)		
Iengar et al. [51]	50	71	60 Gy	79.5%	86.6%	26.3%	37.7% (1-y)	17% gr. 3–5	8.7 m
			4 Gy/fr					4% gr. 5	
Franceschini et al. [52]	41	78.6	50–56 Gy	76%	NS	49%	51.3%	No	9.9 m
			2.5–2.8 Gy					(1-y)	
Kravutski et al. [53]	76	76.7	38–56 Gy	NS	NS	NS	67%	5.2% gr. 3	46.8 m
			2.5–3.8 Gy/fr					(1-y)	
Hopkins et al. [54]	41	73	60 Gy	NS	NS	NS	9 m	No	12.2 m
			4 Gy/fr				(median)		
Valeriani et al. [55]	76	70	60 Gy	NS	NS	60.6%	38.9%	3.9% gr. 3	50 m
			3 Gy/fr				(2-y)		
Eze et al. [56]	47	72	42–49 Gy	NS	NS	29.8%	66%	4.2% gr. 3	28.9 m
			2.8–3.5 Gy/fr				(1-y)		
Tekatli et al. [57]	47	77.5	60 Gy	100%	98%	30%	20.1%	38% gr. 3–5	29.3 m
			5 Gy/fr				(3-y)	15% gr. 5	

LC: local control; RC: regional control; DM: distant metastases; Gy: gray; m: months; NS: not specified.

**Table 6:** Proposed protocol combining immunotherapy and hypofractionated radiotherapy for older patients with locally advanced non-small cell lung cancer.

PD-L1 < 1	60 Gy in 3 Gy/fraction followed by 8 cycles of immunotherapy every three weeks [24,25,26,27,55]
PD-L1 = 1 or more	4 cycles of immunotherapy first followed by 60 Gy in 3 Gy/fraction. Immunotherapy will resume after radiotherapy for four cycles unless excessive toxicity observed during the induction phase [17,39,40,41,42,55].

PD-L1: programmed death ligand 1.

## Data Availability

The data presented in this study are available in this article.
